# Prototype for rapid test devices to detect residues of sulfonamides in chicken carcasses from traditional breeders in Surabaya, Indonesia

**DOI:** 10.14202/vetworld.2023.1252-1259

**Published:** 2023-06-08

**Authors:** Mochamad Lazuardi, Eka Pramyrtha Hestianah, Tjuk Imam Restiadi

**Affiliations:** 1 Sub-Division of Veterinary Pharmacy, Faculty of Veterinary Medicine, Universitas Airlangga, Mulyorejo Rd, 60115, Surabaya, Indonesia; 2 Division of Histology, Faculty of Veterinary Medicine, Universitas Airlangga, Mulyorejo Rd, 60115, Surabaya, Indonesia; 3 Division of Reproduction, Faculty of Veterinary Medicine, Universitas Airlangga, Mulyorejo Rd, 60115, Surabaya, Indonesia

**Keywords:** diazotation, food safety, residues, sulfadiazine, veterinary drugs

## Abstract

**Background and Aim:**

Sulfadiazine, one of the sulfonamide group’s active compounds, is widely used for therapeutic production against several diseases. Veterinary drug residues can have a significant impact on human health conditions. This study aimed to develop a prototype of rapid test devices (RTDs) for detecting sulfadiazine residues on chicken carcasses based on the color indication.

**Materials and Methods:**

Seven samples of carcasses collected from traditional breeders in Surabaya-Indonesia were prepared and tested using RTDs. This sample represents the population considering that in the last report, the use of antibiotics was more than 40%, while the ability to monitor RTDs was estimated at 100. The standard color of purple by Hex code standard color or decimal code color was used to compare the positive samples. A light-emitting diode (LED) lamp was used to observe purple color. Analysis of sulfonamides resulting from RTDs was compared using a ultraviolet-visible spectrophotometer.

**Results:**

Sulfonamides contamination levels of 50% and 100% were detected at concentrations of 0.472 μg/mL and 0.642 μg/mL, respectively. Sulfonamides contamination that was <0.395 μg/mL did not appear purple.

**Conclusion:**

The study’s findings showed that RTDs can be used to detect sulfonamides residues at a limit of detection 0.5 mg/mL after a 45 min exposure to an LED operating at a wavelength of 980 nm (p < 0.05). The limitation of RTDs was not being able to monitor the presence of residues bound in fat samples. Rapid test devices can be developed for commonly monitoring devices due to the limited technology available in the market.

## Introduction

According to Sustainable Development Goals 12.3 Food and Waste, potentially dangerous chemical pollutants, such as veterinary medicine residues, must be avoided [1, 2]. In addition, antibiotic residues promote contaminants that are harmful to human health, including the problem of antimicrobial resistance (AMR) [3]. The use of antibiotics before animal slaughter has the highest proportion of AMR risk [4]. The percentage of the medium during the slaughtering process is due to fecal contaminants containing antibiotics that contaminate the meat. The smallest percentage of risk of AMR emergence is when packaging and selling fresh carcasses in the market [3–5]. Currently, one health program tightened the use of antibiotics in livestock whose products will be consumed by humans, such as meat, milk, and eggs [6].

Although chicken is the most popular food worldwide, it could contribute to AMR occurrence. In addition, sulfonamides are a class of drugs particularly useful in treating and preventing bacterial infections in livestock production and veterinary clinics [7]. Sulfonamides have shown an increasing trend in the past three years in poultry farming industries, especially for oral sulfonamides [8, 9]. Dihydropteroate synthase, which condenses pteroate and p-aminobenzoic acid (pABA) to form dihydropteroate through folic acid, is known to be inhibited by sulfonamides. In addition, these compounds also compete with pABA at the active site of the enzyme to act as an alternative substrate and produce pteroate-sulfonamide complex from which the bacteria cannot generate folic acid [10]. It is known that benzene’s aromatic ring structure has a high ionic energy, enabling charge transfer resonance to occur between the bonds between carbon atoms. This characteristic causes sulfonamide compounds to be liposoluble so they can quickly spread to deep body tissues. Sulfonamides in the animal body have a high variability of bioavailability [11, 12]. The main focus of this concern is the availability of sulfonamide residues before the withdrawal period. The risk of residues directly affects people who use carcasses as a source of protein for their animals. If consumers are taught in advance to be cautious when purchasing corpses or to have residue-free tests, this issue will not develop. Furthermore, it is well recognized that no residue quick test devices, rapid test device (RTD), are currently available.

It is well known that the success of the test depends on the presented color substance and is straightforward for humans to observe. Furthermore, the range of observation should be between 400 nm and 700 nm. Meanwhile, it is known that sulfonamides can be monitored under visible wavelength (545 nm) using a ultraviolet (UV)-viable spectrophotometer [13]. The detection method of sulfonamides by UV-visible spectrophotometer is based on color presentation reaction depicts by their compound to produce diazotate bond [14, 15]. The concepts described above were useful to carry over to produce of the rapid test kit to detect the residue of sulfonamides. This can be applied to detect the analyte samples free from impurities. Separation analysis from biological matrixes or impurity compounds is carefully performed to support this idea. The limited matrix biology used for the samples analysis was the main concern of this method. The corpses of chickens have been proposed as a fitting response to the previous explanation of theoretic grand design [16].

The hypothesis of this present study was to develop a prototype for RTDs to detect the residue of sulfonamides. This study determined sulfonamides using a UV-visible spectrophotometer as a comparison. According to the study background, a quick test prototype for sulfonamide residue detection was designed and researched using the diazo bond technique in specific samples detectable in chicken carcasses.

## Materials and Methods

### Ethical approval

This study did not require animal ethic clearance because the carcasses were obtained from local breeders in fresh form.

### Study period and location

This research was conducted from December 2021 to August 2022, using samples from local breeders around Surabaya – Indonesia, at geographical coordinates: 7° 14’ 57” South, 112° 45’ 3” East. All studies were conducted in the Laboratory of Veterinary Pharmacy, Faculty of Veterinary Medicine, Universitas Airlangga. Especially for testing the beam strength of the light source, it was carried out at the Robotics Laboratory of Sepuluh November Institute of Technology Surabaya – Indonesia.

### Reagents and apparatus

Sulfonamides used in this study for analysis were of more than 99.0% purity and purchased from Sigma, USA (catalog Number S8626-25G). All the other chemicals were identified as pure substances at pro analysis levels were purchased from Merck, such as sodium nitrite (1.06549), trichloroacetic acid (607-004-00.7), ammonium amido sulfonate (1220.0100.204L636620), n-(1-naphthyl) ethylene-diamine dihydrochloride (NED) (1.06237.005), hydrochloride acid, water pro chromatographic, and sodium hydroxide.

The chemicals were prepared at full strength as a diazotation reaction at designated concentrations and kept in opaque bottles. The usage of all reagents reached their peak stability after around 3 days. All reagents were prepared as follows; sodium nitrite 0.15% in water for chromatographic level (w/v), trichloroacetic acid 15% in water for chromatographic level (w/v), ammonium amido sulfonate 0.75% in water for chromatographic level (w/v), NED 0.17% in water for chromatographic level (w/v), sodium hydroxide 10% in water for chromatographic level (w/v), and hydrochloride acid 0.1 N in water for chromatographic level (v/v). The sulfadiazine was made into stock solutions at a concentration of 100 g/mL using water that was free of CO_2_ and sodium hydroxide was added in small amounts to aid in the sulfadiazine’s dissolution in water. At the Faculty of Veterinary Medicine, Universitas Airlangga, the Veterinary Pharmacy Laboratory, all reagent preparation was carried out in a clean environment at 22°C. Utilizing a magnifying glass type Newmark EU-2038 made by Zhejiang Semtom Electronic Co., Ltd. in China, the counterfeit money detector used light-emitting diode (LED) lamps with wavelengths ranging from red (at a wavelength of roughly 700 nm) to blue-violet (about 400 nm). The Genesys 10S UV-visible spectrophotometer, manufactured by ThermoFisher Scientific in the UK, was used in photometric milliabsorbance units (mAU) or absorbance unit (AU) mode.

### Research design

The research design used an experimental model with control groups and samples of chicken carcasses under sulfadiazine contamination.

### Sample test

Seven samples were used for the chicken carcass test as shown in Table-1. According to reports, the food safety and security committees in each nation screened 40% of the population, and all samples revealed residues [17]. In addition, the determination of the number of chicken populations in Table-1 was also based on the findings of a 2017 study in Indonesia using antibiotics in chickens around 4.17%–83.3% [16, 18]. Thus, if half of the population was taken, 40% would be obtained. If the RTD is able to monitor to high sensitivity, 100% of the antibiotic residue will be found [18]. Carcasses without number control from veterinarians were procured from seven areas in Surabaya, Indonesia, using proportional randomization from traditional breeders. A local traditional breeder must only raise chickens for use as pets and must have no more than 7–10 of them. The following locations for the carcasses sampling were noted: One sample from Surabaya’s north, two samples from its west, two samples from its east, and two samples from its south. The carcass samples were powdered and put in sulfadiazine stock solution for 48 h, then 5 g of each sample was taken. In a syringe barrel without a plunger or needle, each 5 g sample was placed. In addition, samples were forced with a plunger lower into the barrel’s bottom to get crushed and readily pass through the adapter hole. After that, the samples were placed in a 2.5 mL tube and stored at 20°C until required.

**Table-1:** Determination of sample size by an error in the population (α) and error in the sample (β) at 5%.

Prediction found the residue in poultry carcasses (%)										Prediction found the residue in population (%)

10	20	30	40	50	60	70	80	90	100	
53	28	17	12	9	7					0
	270	83	42	26	18	13	9	7		10
		402	111	53	31	20	14	9		20
			294	128	58	32	20	13		30
				539	134	58	31	18	7	40
					539	128	53	26	9	50
						494	111	42	12	60
							402	83	17	70
								270	26	80
									53	90

### Scanning for a specific wavelength and diazotation

**Figure-1:**
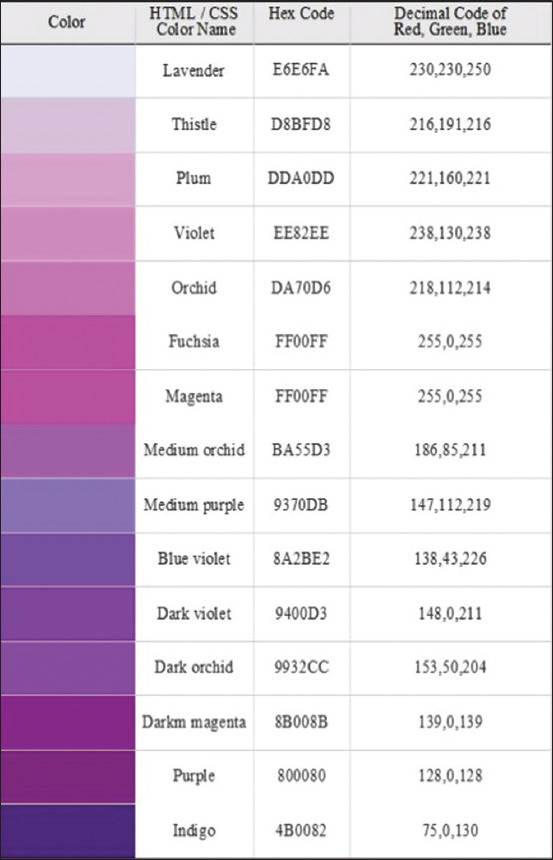
Purple color chart on hypertext markup language or cascading style sheets, hex code, and decimal code.

Stock solutions of sulfadiazine were adjusted at 15^th^ series dilutions ranging from 0.1 to 100 μg/mL using three replicates of each concentration, including 0.1, 0.5, 1, 5, 10, 15, 20, 30, 40, 50, 60, 70, 80, 90, and 100 mg/mL. All concentrations were diazotation and described as follows, and the displayed color was seen using a UV-visible spectrophotometer. Forty-five test tubes (5 mL) were arranged in a tray. The test tubes were filled with 500 mL of each concentration in three replications (N1, N2, and N3), 500 mL of water mixed and shaken immediately. To this solution, 500 μL of trichloroacetic was added. The solution was centrifuged at 1500× *g* for 10 min and 200 μL supernatant was taken from each concentration and placed in new serials test tubes. Each analyte received an initial addition of 100 mL of sodium nitrite, followed by an instantaneous addition of 200 mL of ammonium amido sulfonate and shaking. In addition, 200 L of NED was gradually added to each tube until a purple color appeared due to diazo reactions. The criteria of purple color have been presented as five-tenth Red Green Blue (RGB) Hex or decimal code of 218,112,214–138,43,226 (Figure-1). The final phase involved the addition of stabilization reagents while simultaneously adding 3.8 mL of CO_2_-free water and 200 μL of HCl acid (pH 0.93–1.00).

In addition, the analyte concentration of 0.1 g/mL was measured using a UV-visible spectrophotometer in photometric mode with a detection device set up at a wavelength between 540 and 550 nm (scaling interval 2 nm). After scanning the selected wavelength, the wavelength was used for further observations. Next, the stability of the solution was assessed using the amount of time spent at a fixed wavelength with a steady absorbance at a 0.1 g/mL analyte concentration. The additional evaluation compares the relationship response detector’s intraday accuracy and percent coefficient variation (%CV) of the absorbance (mAU) to serial concentrations. The system-suitable test of relationship response detector versus serial concentration and the intraday precision of R^2^ at ≥0.90, and not more than <3% of CV for intraday precision was the criteria for the test. The UV-visible spectrophotometer method’s limit of detection (LOD) was developed with lower concentrations of analyte without absorbance from impurities agent, followed by the creation of a linear equation, y = a + bx, with subsequent concentrations up to the lowest concentration. The value of x at the lowest concentration was LOD, furthermore the 3 times of LOD, namely, limit of quantification (LOQ).

### Research procedures

Three groups of tests containing seven samples each were evaluated as follows; Group 1 served as a sample of containment carcasses, Group 2 as a carcass devoid of sulfadiazine residues, and Group 3 as a stock solution for sulfadiazine. Group 1 was ready, as stated in the subsequent phase. Using the barrel of a disposable syringe without a needle, 5 g of carcass flesh was pulverized and crushed.

**Figure-2:**
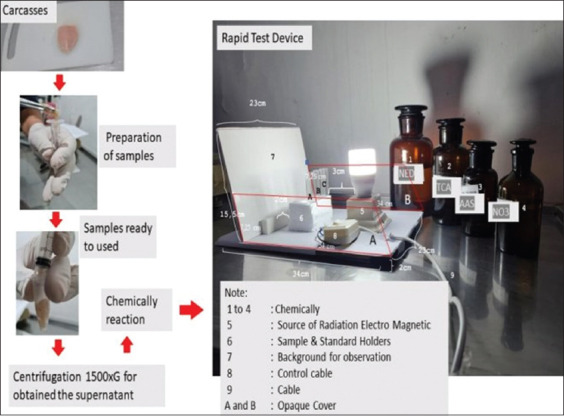
The sample preparation process and ready to use for observation using rapid test devices of sulfonamides.

The preparation of fine meat was then achieved by pressing the syringe with a plunger so that the gasket force the meat out. Furthermore, the shelter was carried out in a 3 mL tube, given 2–3 drops of distilled water, and then centrifuged at 1500× *g* for 30 min (Figure-2). The supernatants were carefully removed and prepared for use as a test sample.

The processed diazotation related to points 1–8 was presumed to be present in the supernatants. The plasma’s purple color was compared to the purple color chart in Figure-1. Group 2 received the same treatment as Group 1. Sulfonamides were added to group 3 at concentrations of 0.3, 0.4, 0.5, 0.6, and 0.7 μg/mL. The Group 3 was then processed similarly to Group 1 at points 1–8 to obtain the color indication shown in Figure-1. The determination of LOD and LOQ for rapid tests was proceed, referring to the modified method as the same using a UV-visible spectrophotometer with the following steps. The lowest response point value was then obtained using probit analysis.

### Statistical analysis

The Statistical Package for Social Sciences 24.0 software (IBM Corp., NY, USA) was used to assess R^2^ of C_1–15_ versus A_454_. Limit of detection for quick test device in probability 50% observed and hypothesis test for significantly different three independent objects as samples as opposed to positive control and negative control using one-way analysis of variance test (p < 0.05).

## Results

**Figure-3:**
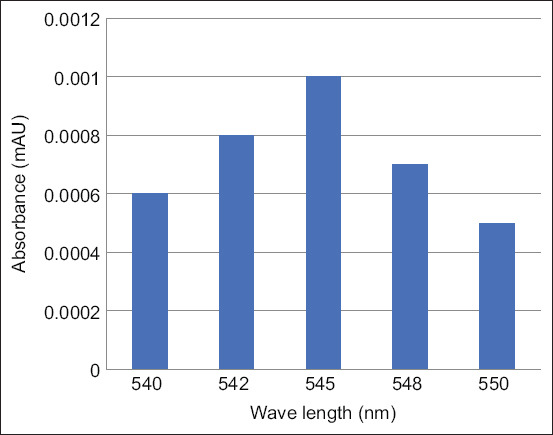
Scanning photometric profile wave length versus absorbance of sulfadiazine 0.1 μg/mL in solvent comprised of sodium nitrite 0.15%, trichloroacetic acid 15%, ammonium amido sulfonate 0.75%, n-(1-naphthyl ethylene-diamine dihydrochloride 0.17% and hydrochloric acid 0.1 N at pH 0.9.

**Figure-4:**
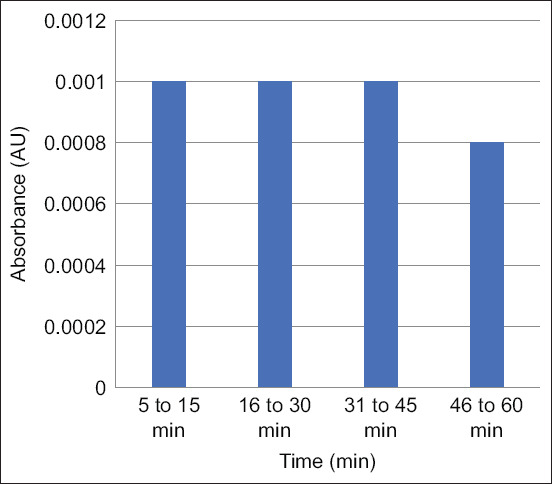
According to an analysis, sulfadiazine at 0.1 g/mL depicts a steady absorbance for 45 min at 545 nm.

The selected wavelength was 545 nm for the analyte solution in the pH range of 0.97–1.20 (Figure-3), and the length of time that showed a stable absorbance was 45 min since the last addition of 0.1 N HCl and water free from CO_2_ (Figure-4). The results of linearity and intraday precision are shown in Table-2.

**Table-2:** Analysis relationship between concentrations versus response detector and intraday precision sulfonamide solution.

No.	Concentrations of C_1–15_ (μg/mL)				Absorbance of A_545_ (AU)			
	
	N_1_	N_2_	N_3_	Mean ± SD	N_1_	N_2_	N_3_	Mean ± SD
1	0.101	0.102	0.102	0.102 ± 0.001	0.001	0.002	0.003	0.002 ± 0.001
2	0.501	0.500	0.502	0.501 ± 0.001	0.005	0.004	0.006	0.005 ± 0.001
3	1.012	1.011	1.013	1.012 ± 0.001	0.011	0.012	0.014	0.012 ± 0.001
4	5.001	5.002	5.004	5.002 ± 0.001	0.052	0.054	0.049	0.052 ± 0.002
5	10.001	10.003	10.002	10.002 ± 0.001	0.112	0.114	0.113	0.113 ± 0.001
6	15.002	15.001	15.002	15.002 ± 0.001	0.165	0.163	0.167	0.165 ± 0.003
7	20.003	20.002	20.001	20.002 ± 0.001	0.224	0.226	0.227	0.226 ± 0.001
8	30.011	30.012	30.001	30.008 ± 0.006	0.339	0.337	0.336	0.337 ± 0.001
9	40.000	40.001	40.002	40.001 ± 0.001	0.451	0.450	0.455	0.452 ± 0.001
10	50.011	50.012	50.012	50.012 ± 0.001	0.564	0.563	0.566	0.564 ± 0.001
11	60.012	60.011	60.013	60.012 ± 0.001	0.682	0.681	0.679	0.681 ± 0.001
12	70.014	70.012	70.011	70.012 ± 0.002	0.830	0.829	0.834	0.831 ± 0.003
13	80.018	80.017	80.019	80.018 ± 0.001	0.932	0.931	0.933	0.932 ± 0.001
14	90.011	90.010	90.013	90.011 ± 0.002	1.011	1.014	1.012	1.012 ± 0.002
15	100.001	100.002	100.004	100.002 ± 0.001	1.122	1.126	1.124	1.124 ± 0.002

**Figure-5:**
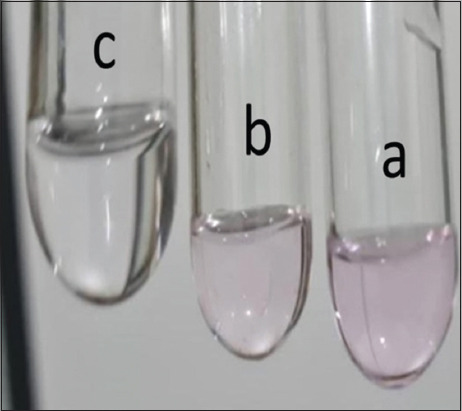
(a) Carcasses sample with sulfonamide (0.5 μg/mL) contamination, (b) positive control containing 0.5 μg/mL of sulfonamides, and (c) negative control.

Analysis relationship between C_1–15_ and A_545_ indicated a strong relationship between concentrations and absorbance at R^2^ 0.99. Table-2 illustrates the precision of the %CV weighing for C. Limit of detection A_545_ was found at 0.05 μg/mL (0.0004 AU) a concentration of sulfonamide, then by producing a regression equation for concentrations of 0.002 μg/mL (0.0003 AU) and 0.001 μg/mL (0.0001 AU) obtained the equation y=9.10^−5^+0.06x, then the LOD is 0.008 μg/mL at minimum absorbance of 0.0006 AU. The LOQ at 3 times of LOD was a sulfonamides solution of 0.024 μg/mL. Table-3 displays the data analysis for the computed LOD for the rapid test sulfonamides. If the LOD is detected at a concentration of 0.5 g/mL, there is a 50% probability that the test will be applied successfully. Figure-5 displays a positive test result at a sample residue concentration of 0.5 g/mL.

**Table-3:** Rapid test of sulfonamides in seven poultry carcasses.

Concentrations (μg/mL)	Groups 1 Samples (±)	Group 2 Negative control (±)	Group 3 Positive control (±)	p-value
0.300	0/7	0/7	7/0	
0.400	1/6	0/7	7/0	<0.05
0.500	4/3	0/7	7/0	
0.600	7/0	0/7	7/0	
0.700	7/0	0/7	7/0	

According to research findings (Table-4), sulfonamide contaminants at the concentrations of 0.472 μg/mL and 0.642 μg/mL were determined to be 50% and 100% effective.

**Table-4:** Comparison between rapid test devices and similar test devices in the market.

Scope	Rapid test devices	Other similar devices
Process	Easy	Moderate to complicate
Cost	Not too expensive	Not too expensive
Effective	Usefully and not	Depends on sample requirements and temperature control of the testing room
Specificity	Specific to sulfonamides derivatives	Can be used to test other sulfonamides as an antibiotic
Safety	Safe for operators, the waste product not influenced the environment	Safe for operators, the waste product not influenced the environment

Contaminant with <0.395 μg/mL of sulfonamides did not show purple color as observed by human eyes (Figure-5, point C). The strongest color shown in point A of Figure-5 indicated that as compared to the negative control, contaminant was observed at more than 0.472 μg/mL (Figure-5, point B). When normalized and homogenized data were used in the hypothesis test for the groups samples versus control negative groups and control positive groups shown in Table-3, the results showed a significant difference between the samples (p < 0.05).

## Discussion

All chemicals used in this study were of analytical and chromatographic grade with absolutely zero contaminant levels. The high purity standards ensured that diazo reactions were carried out smoothly [19]. Table-1 shows the sample size criteria, with a population error rate (α), sample error rate (β) of 0.05, and carcass number of 7.0. This method is very practical for obtaining a minimalist test sample with a small error rate [17, 18]. If the organ parts are found to have rather substantial residual contamination, the carcass parts are obtained directly using a modified grab sample technique [19–22]. A total of 5 g of carcass samples using the preparation technique described above basically uses a method that has been developed to test for tetracycline residues in chicken carcasses [5, 16, 23, 24]. Overview of the sample preparation techniques carried out in this study is quite simple and fast. However, it requires a pre-requisite for sample acquisition in preparation, namely, the level of clarity of the supernatant after centrifugation of some samples. The clearer the supernatant results, the less the component contamination from the carcass matrix which often disrupts the diazotizes bonds [25–27]. This RTD device’s weakness is that the purple color that results from the diazotized bond soon fades when sunshine breaks down the bond. Therefore, adding the NED solution in a dark place is necessary. In an open space between 20°C and 25°C with 20%, the stability of color appearance at a wavelength of 545 nm was lasted 45 min (Figure-4). A comparison study between RTDs and UV-visible spectrophotometers revealed that the detection capability of RTDs devices is 20 times lower than that of UV-visible spectrophotometers [28]. UV-visible spectrophotometers detected at small concentrations were linear with good precision at a %CV <3.0 as shown at Table-2. This investigation demonstrated that the concentration range provided in Table-2 for the UV-visible spectrophotometer examination using an optical type detector could still be monitored by a diazotization principle test instrument [29].

In an acidic media, the diazotization reaction occurs between nitrite and a chemical containing primary aromatic amines to produce a diazonium salt. Some compounds with primary aromatic amines commonly used as a source of diazonium salts are aniline, sulfanilic acid, or ρ-nitro aniline. Sulfonamides have an aromatic ring structure containing nitrogen ions. Diazotization creates bonds between N ions, making the double bonds easily identified as a sulfonamide. N-(1-naphthyl) ethylene-diamine dihydrochloride increases the visibility of the diazo bonds, making the indicator’s transition to a purple color more apparent [30].

Table-3 shows the appearance of purple color appeared above the LOD concentration. The color became weak when electromagnetic radiation was stimulated from the sun’s light source. The usage of RTDs is thus advised in this study to be free of electromagnetic radiation from sunlight sources. This investigation has shown several flaws with comparable RTDs that have been commercially available (Table-4 and Figure-4).

In Figure-5, it was observed that the purple color of the samples containing sulfonamide (A) was almost equivalent to the purple color of the positive control (B). This would have been more obvious if monitored on RTDs with a white background and a light source from LED lights. This color can also be specified using the standard purple color criteria in the decimal code five-tenth RGB Hex. With the emergence of the LED light source and the contribution of the cover opaque on both sides of the RTDs as presented in Figure-2, the neutral hue depicted in Figure-5, point C was more distinct, and with normal vision, the colors in tubes point A and point B may be identified.

## Conclusion

In this study, it can be concluded that RTDs can be used to determine the carcasses’ contamination with sulfonamides. Color observation on RTDs is more perfect than observation by the presence of an opaque cover on both sides. The main design guiding principles for RTDs were quick, simple, affordable, efficient, sturdy, and safe. The purple color often only appears for 45 min on average, and even then, the concentration has to be higher than 0.5 μg/mL. The addition of NED should be done in a dark room to provide the best workmanship, which is a suggestion for using RTDs. The limitation of RTDs was not being able to monitor the presence of residues bound in fat samples. Rapid test devices can be developed for common monitoring devices due to the limited technology available in the market.

## Authors’ Contributions

ML: Conceived and designed the experiments, performed the experiments, contributed reagents, materials, analysis tools and data. ML, EPH, and TIR: Analyzed and interpreted the data, and wrote the manuscript. All authors have read, reviewed, and approved the final manuscript.
